# Comparison of carotid atherosclerotic plaque characteristics between symptomatic patients with transient ischemic attack and stroke using high-resolution magnetic resonance imaging

**DOI:** 10.1186/s12872-022-02624-7

**Published:** 2022-04-21

**Authors:** Mingming Lu, Lichen Zhang, Fei Yuan, Peng Peng, Hongtao Zhang, Shitong Liu, Yao He, Jianming Cai, Xihai Zhao

**Affiliations:** 1 Institute of Geriatrics, State Key Laboratory of Kidney Disease, Beijing Key Laboratory of Aging and Geriatrics, The Second Medical Center, Chinese PLA General Hospital, Beijing, 100853 China; 2 Department of Radiology, Pingjin Hospital, Characteristic Medical Center of Chinese People’s Armed Police Force, Tianjin, China; 3 Department of Radiology, The Fifth Medical Center, Chinese PLA General Hospital, Beijing, 100853 China; 4 Center for Biomedical Imaging Research, Department of Biomedical Engineering, Tsinghua University School of Medicine, Beijing, China

**Keywords:** Carotid artery, Atherosclerosis, Stroke, Transient ischemic attack, Magnetic resonance imaging

## Abstract

**Background:**

This study aimed to compare the characteristics of carotid plaques between patients with transient ischemic attack (TIA) and ischemic stroke using magnetic resonance (MR) imaging.

**Methods:**

Patients with a recent ischemic stroke or TIA who exhibited atherosclerotic plaques of carotid arteries in the symptomatic sides determined by MR vessel wall imaging were recruited. The plaque morphology and compositions including intraplaque hemorrhage (IPH), lipid-rich necrotic-core (LRNC) and calcification were compared between TIA and stroke patients. Logistic regression was performed to relate the plaque characteristics to the types of ischemic events.

**Results:**

A total of 270 patients with TIA or ischemic stroke were recruited. Stroke patients had a significantly higher prevalence of diabetes (42.2% vs. 28.2%, *p* = 0.021), greater mean wall area (35.1 ± 10.1 mm^2^ vs. 32.0 ± 7.7 mm^2^, *p* = 0.004), mean wall thickness (1.3 ± 0.2 mm vs. 1.2 ± 0.2 mm, *p* = 0.001), maximum normalized wall index (NWI)(63.9% ± 6.0% vs. 62.2% ± 5.9%, *p* = 0.023) and %volume of LRNC (9.7% ± 8.2% vs. 7.4% ± 7.9%, *p* = 0.025) in the carotid arteries compared to those with TIA. After adjustment for clinical factors, above characteristics of carotid arteries were significantly associated with the type of ischemic events. After further adjustment for maximum NWI, this association remained statistically significant (OR, 1.41; CI, 1.01–1.96; *p* = 0.041).

**Conclusions:**

Ischemic stroke patients had larger plaque burden and greater proportion of LRNC in carotid plaques compared to those with TIA. This study suggests that ischemic stroke patients had more vulnerable plaques compared to those with TIA.

## Introduction

Stroke has become the leading cause of death and disability in the Chinese population [1] and it has been well established that carotid atherosclerotic disease is one of most important risk factors of ischemic strokes, contributing to up to 20–30% of strokes or transient ischemic attack (TIA) [2, 3]. A previous study reported that patients with ischemic stroke had a higher risk of recurrent ischemic events compared to patients with TIA (11.5% vs. 5.2%) [4]. However, the potential mechanisms for the different recurrent risk of ischemic events between carotid atherosclerotic patients with TIA and stroke remained unknown. Therefore, it is important to clarify the differences in carotid plaque characteristics which may be related to the recurrence of cerebrovascular events between patients with TIA and stroke.

The differences in carotid plaque characteristics between symptomatic and asymptomatic patients have been fully studied [5, 6], however, few studies have compared the differences between plaque features from ischemic stroke and TIA patients. A previous study [7] showed that stroke patients had increased leaky plaque microvasculature compared to those with TIA, suggesting that microvasculature in plaques may play an important role in the occurrence of different ischemic events. In addition to microvasculature in plaques, more vulnerable plaque features including greater plaque burden, intraplaque hemorrhage (IPH), larger lipid-rich necrotic-core (LRNC) and ruptured fibrous cap might be more closely associated with the severity of cerebrovascular ischemic events. However, it remains unclear whether the carotid plaque vulnerability varies between patients with TIA and ischemic stroke. Multi-contrast magnetic resonance imaging (MRI) has been proven to have excellent value in characterizing morphological and compositional features of carotid plaque in symptomatic patients [8, 9]. Thus, this study aimed to compare the characteristics of carotid plaques between patients with TIA and ischemic stroke using the multi-contrast MR vessel wall imaging.

## Materials and methods

### Study population

Patients who suffered from cerebrovascular ischemic symptoms including recent stroke or TIA and exhibited atherosclerotic plaques of carotid arteries in the symptomatic sides determined by MR vessel wall imaging were consecutively recruited in this study. Patients with the following conditions were excluded:1) no confirmed side of symptom; 2) presence of a possible cardiac embolic source such as atrial fibrillation; 3) symptomatic carotid artery with > 70% of stenosis; and 4) a history of ischemic stroke in patients with recent TIA. Patients only with confirmed symptomatic side of TIA/stroke and exhibited ipsilateral carotid plaques were recruited to ensure the carotid atherosclerosis as the true culprit lesions of ischemic events as much as possible. TIA was defined as the rapidly developing signs of a neurological deficit or monocular loss of vision, lasting less than 24 h with no apparent cause other than that of vascular origin. Ischemic stroke was defined as the rapidly developing clinical signs of a neurological deficit, lasting more than 24 h with no apparent cause other than that of vascular origin and without evidences of an intracranial hemorrhage on CT/MR images [7]. The clinical information of all patients, such as age, sex, body mass index (BMI), hypertension, diabetes, hyperlipidemia, smoking, anti-hypertension medication use, antiplatelet agent use and statin use, was collected and reviewed. The study protocol was performed in accordance with the Declaration of Helsinki and was approved by institutional review board of Chinese PLA General Hospital (No.20010006) and all subjects provided written informed consent form.

### Carotid artery MR imaging

All MR imaging was performed on a 3.0 T MR scanner (SignaHDx, GE Medical System, Milwaukee, WI, USA) with dedicated phase-arrayed carotid coils. Multi-contrast MR protocol including following parameters: three-dimensional (3D) time-of-flight (TOF) MR angiography: repetition time (TR)/echo time (TE) 29 ms/4.9 ms, field of view (FOV)14 × 14 cm^2^,matrix size 256 × 256, flip angle 20°, and 2 mm slice thickness; T1-weighted (T1W) quadruple inversion recovery (QIR): TR/TE 800 ms/10 ms, FOV14 × 14 cm^2^,matrix size 256 × 256, flip angle 90°, and 2 mm slice thickness; and T2-weighted (T2W) multi-slice double inversion recovery (DIR): TR/TE 4800 ms/50 ms, FOV14 × 14 cm^2^,matrix size 256 × 256, flip angle 90°, and 2 mm slice thickness. All MR imaging was centered to the bifurcation of the symptomatic side of the carotid artery with longitudinal coverage of 24 mm.

### MR image analysis

Two radiologists with more than 3 years’ experience in plaque imaging interpreting reviewed the MR vessel wall images of carotid arteries using custom-designed software CASCADE (University of Washington, Seattle, USA) [10] and were blinded to clinical information of all recruited patients. The quality of carotid MR images was graded as poor, marginal, good and excellent according to the overall signal-to-noise and images graded as poor were excluded from this study. The lumen and wall boundaries were traced manually and the lumen area, wall area, maximum wall thickness, and wall volume was measured and calculated. Measure of carotid plaque burden was expressed by the normalized wall index (NWI) which was defined as the wall area divided by the total wall area. The degree of luminal stenosis of carotid arteries was measured on the 3D TOF MR angiographic images by maximum intensity projection reconstruction according to the North American symptomatic carotid endarterectomy trials criteria [11]. The presence or absence of plaque compositions, such as lipid-rich necrotic core, intraplaque hemorrhage and calcification was identified and the volume of each plaque component as a percentage of the wall volume was measured and calculated according to published criteria [12, 13]. The wall volume was calculated by 2 mm × wall areas of slice with plaque.

### Reproducibility

Twenty patients were randomly selected from the study sample for testing the inter-observer and intra-observer agreement in measuring morphology and compositions of carotid plaque. All the patients were used for testing the inter-observer and intra-observer agreement in identifying the presence of carotid plaque compositions.

### Statistical analysis

Continuous variables were summarized as mean ± standard deviation (SD) and categorical variables were presented as percentage. The clinical characteristics and carotid plaque measurements were compared between patients with TIA and stroke using independent Student *t*-test, Mann–Whitney U test or chi-square test. Univariate and multi-variable logistic regression models were used to estimate the odds ratio (OR) and corresponding 95% confidence interval (CI) of carotid plaque features in discriminating the type of ischemic events. Two-sided *p* < 0.05 was considered statistically significant. All the statistical analyses were performed using SPSS 22.0 (IBM, Chicago, IL).

## Results

A total of 379 symptomatic patients with TIA or ischemic stroke were recruited. Of the recruited 379 patients, 109 patients were excluded due to the following reasons: 1) poor image quality (12 patients); 2) no confirmed side of ischemic symptoms (81 patients); 3) insufficient longitudinal coverage (16 patients). Of the remaining eligible 270 patients, 142 patients (mean age: 64.3 ± 10.4 years old; males: 66.9%) diagnosed with TIA and 128 patients with ischemic stroke (mean age: 66.1 ± 9.2 years old; males:75%) were finally included for statistical analysis.

### Clinical characteristics

Among the 270 included symptomatic patients, the prevalence of the patients with hypertension, hyperlipidemia, diabetes was 79.6%, 58.1%, and 34.8%, respectively. Additionally, 140 (51.9%) patients had a history of smoking, 185 (68.5%) had anti-hypertension medication use, 166 (61.5%) had antiplatelet agent use and 115 (42.6%) had statin use. Stroke patients had significantly higher prevalence of diabetes (42.2% vs. 28.2%, *p* = 0.021) compared to those with TIA. No statistically significant difference was found in other clinical features between these two groups (*p* > 0.005). The comparison of clinical characteristics between patients with TIA and stroke is summarized in Table 1.

**Table 1:** Clinical characteristics of patients with TIA and stroke

	Mean ± SD or n (%)		*p*
	TIA patients (n = 142)	Stroke patients (n = 128)	
Sex, male	95 (66.9)	96 (75)	0.180
Age, y	64.3 ± 10.4	66.1 ± 9.2	0.517
BMI kg/m^2^	23.9 ± 2.9	24.5 ± 3.3	0.083
Hypertension	109 (76.8)	106 (82.8)	0.230
Hyperlipidemia	80 (56.3)	77 (60.1)	0.539
Diabetes	40 (28.2)	54 (42.2)	0.021
Low density protein, mmol/L	3.0 ± 0.9	2.9 ± 0.9	0.200
High density protein, mmol/L	1.1 ± 0.2	1.1 ± 0.3	0.456
Total density protein, mmol/L	1.8 ± 0.9	1.8 ± 1.1	0.609
Triglyceride, mmol/L	4.7 ± 1.0	4.7 ± 1.1	0.964
Statin use	57 (40.1)	58 (45.3)	0.460
Antihypertension medication	94 (66.2)	91 (71.1)	0.432
Antiplatelet agent	81 (57.0)	85 (66.4)	0.133
Smoking	68 (47.9)	72 (56.2)	0.181

SD: standard deviation, BMI: body mass index

### Carotid plaque morphology

**Fig. 1:**
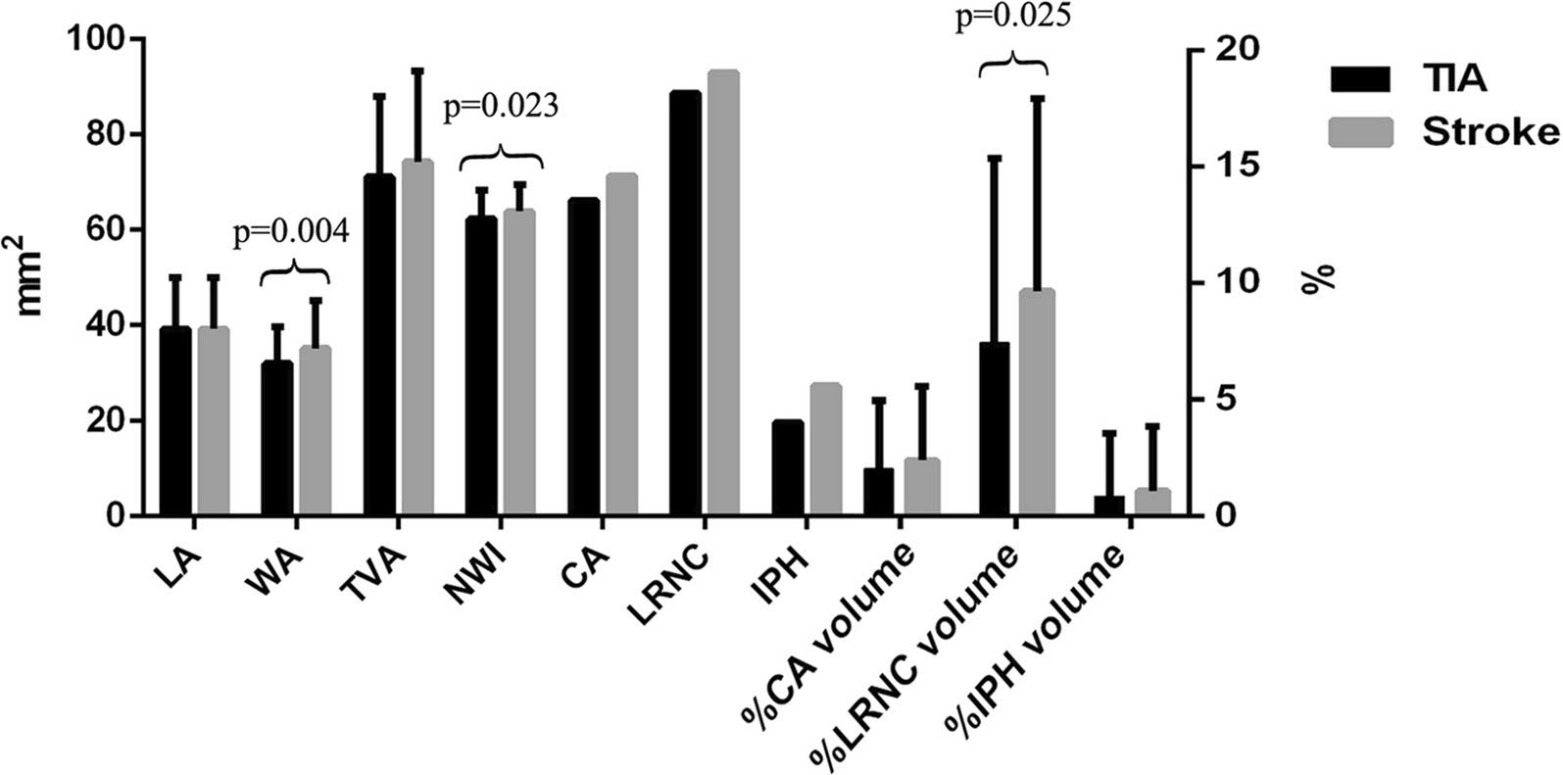
A bar graph for comparison of plaque characteristics of symptomatic arteries between patients with TIA and stroke. LA, lumen area; WA, wall area; TVA, total vessel area; NWI, normalized wall index; CA, calcification; LRNC, lipid-rich necrotic core; IPH, intraplaque hemorrhage; %CA volume, % calcification volume; %LRNC volume, % lipid-rich necrotic core volume; %IPH volume, % intraplaque hemorrhage volume. P values were shown in the picture only if those were less than 0.05

Compared with TIA patients, those with stroke had significantly greater mean wall area (35.1 ± 10.1 mm^2^ vs. 32.0 ± 7.7mm^2^, *p* = 0.004), mean wall thickness (1.3 ± 0.2 mm vs. 1.2 ± 0.2 mm, *p* = 0.001), and maximum NWI (63.9 ± 6.0% vs. 62.2 ± 5.9%, *p* = 0.023) in the carotid arteries (Table 2, Fig. 1). Univariate analysis showed that the mean wall area (OR, 1.04; CI, 1.01–1.07; *p* = 0.005), mean wall thickness (OR, 6.53; CI, 2.07–20.57; *p* = 0.001), and maximum NWI (OR, 1.61; CI, 1.06–2.44; *p* = 0.024) of the carotid artery were significantly associated with the types of ischemic events. Multivariate analysis showed that the association of carotid mean wall area (OR, 1.04; CI, 1.01–1.07; *p* = 0.018), mean wall thickness (OR, 5.52; CI, 1.65–18.45; *p* = 0.006), maximum NWI (OR, 1.56; CI, 1.02–2.38; *p* = 0.038) and the types of ischemic events remained statistically significant after adjusting for age, sex, BMI, and diabetes (Table 3).

**Table 2:** Comparison of carotid plaque characteristics between patients with TIA and stroke

	Mean ± SD or n (%)		*p*
	TIA patients (n = 142)	Stroke patients (n = 128)	
*Plaque morphology*			
Mean lumen area, mm^2^	39.1 ± 10.8	34.2 ± 10.8	0.263
Mean wall area, mm^2^	32.0 ± 7.7	35.1 ± 10.1	0.004
Mean wall thickness, mm	1.2 ± 0.2	1.3 ± 0.2	0.001
Stenosis, %	43.9 ± 9.4	46.5 ± 12.4	0.057
Wall volume, mm^3^	991.3 ± 260.9	1051.1 ± 324.3	0.095
Maximum NWI, %	62.2 ± 5.9	63.9 ± 6.0	0.023
*Presence of plaque components*			
Calcification	94 (66.2)	90 (71.4)	0.514
Lipid-rich necrotic core	126 (88.7)	119 (93.0)	0.294
Intraplaque hemorrhage	28 (19.7)	35 (27.3)	0.152
*% Volume of plaque components*			
Calcification, %	2.1% ± 2.9%	2.4% ± 3.1%	0.345
Lipid-rich necrotic core, %	7.4% ± 7.9%	9.7% ± 8.2%	0.025
Intraplaque hemorrhage, %	0.8% ± 2.7%	1.1% ± 2.8%	0.444

SD: standard deviation, NWI: normalized wall index

**Table 3:** Association between carotid plaque characteristics and the type of ischemic events

	Stroke subtype of ischemic events _a_					
	Univariate model		Multivariate model 1_b_		Multivariate model 2_c_	
	OR (95%CI)	*p*	OR (95%CI)	*p*	OR (95%CI)	*p*
*Morphology of carotid plaque*						
Mean lumen area	0.99 (0.98–1.03)	0.265	0.98 (0.97–1.02)	0.201	-	-
Mean wall area	1.04 (1.01–1.07)	0.005	1.04 (1.01–1.07)	0.018	-	-
Mean WT	6.53 (2.07–20.57)	0.001	5.52 (1.65–18.45)	0.006	-	-
Stenosis	1.02 (0.99–1.04)	0.058	1.02 (0.99–1.04)	0.059	-	-
Wall volume	1.00 (1.00–1.00)	0.097	1.00 (1.00–1.00)	0.252	-	-
Maximum NWI	1.61 (1.06–2.44)	0.024	1.56 (1.02–2.38)	0.038	-	-
*Components of carotid plaque*						
Presence of LNRC	1.68 (0.72–3.95)	0.234	1.61 (0.67–3.86)	0.287	1.47 (0.61–3.57)	0.389
Presence of IPH	1.53 (0.87–2.70)	0.140	1.43 (0.79–2.59)	0.239	1.22 (0.66–2.27)	0.519
Presence of Ca	1.21 (0.72–2.02)	0.469	1.08 (0.62–1.87)	0.792	1.02 (0.58–1.78)	0.944
%Volume of LNRC _d_	1.52 (1.11–2.07)	0.007	1.51 (1.10–2.07)	0.011	1.41 (1.01–1.96)	0.041
%Volume of IPH _d_	1.41 (0.59–3.39)	0.444	1.19 (0.48–2.97)	0.711	0.97 (0.38–2.47)	0.952
%Volume of CA_d_	1.47 (0.66–3.29)	0.345	1.31 (0.56–3.06)	0.540	1.00 (0.41–2.46)	0.994

^a^TIA and ischemic stroke were respectively coded as 0 and 1 in the logistic regression analysis

^b^Model 1 was adjusting for age, sex, BMI, and diabetes mellitus

^c^Model 2 contains model 1 with further adjustment for maximum NWI

^d^Increment of 10%; OR: odds ratio; WT: wall thickness; NWI: normalized wall index; LRNC: lipid-rich necrotic-core; IPH: intraplaque hemorrhage; CA: calcification

### Carotid plaque components

**Fig. 2:**
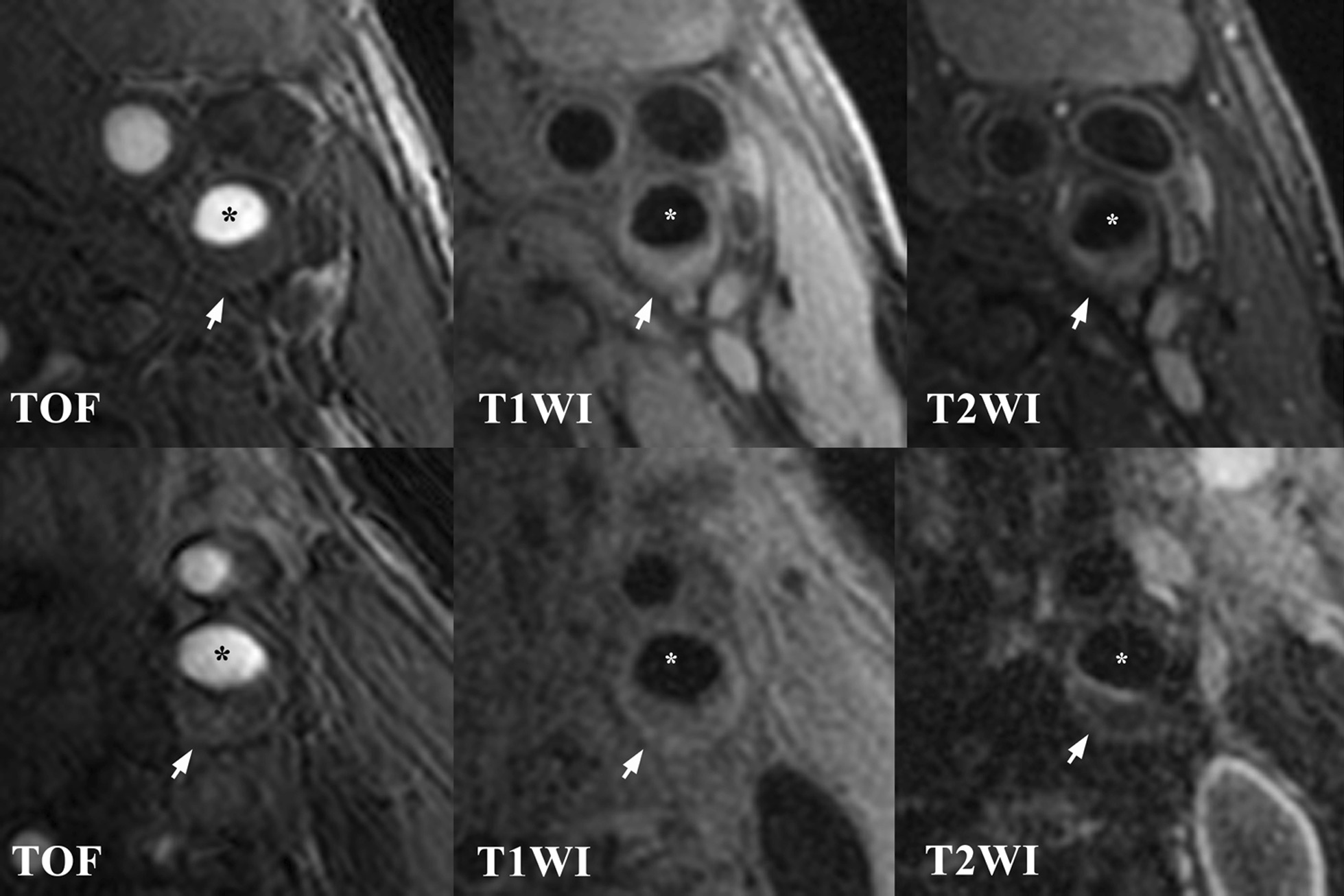
Comparison of carotid atherosclerotic plaques (arrows) between patients with TIA and ischemic stroke. The top row represents a patient (75 years old, male) with recent TIA and the bottom row represents a patient (73 years old, male) with recent ischemic stroke. The images from the bottom row shows carotid plaques with greater proportion of lipid-rich necrotic-core (iso-intense on T1WI and hypointense on T2WI after fat-saturation) compared with those from the top row.*: indicates lumen of internal carotid artery

Patients with stroke had greater %volume of LRNC in the carotid plaque compared with those with TIA (9.7 ± 8.2% vs. 7.4 ± 7.9%, *p* = 0.025) (Fig. 2). However, there were no significant differences in other plaque components including IPH and calcification between these two groups (p > 0.05). Logistic regression analysis showed that %volume of LRNC was significantly associated with the types of ischemic events before (OR, 1.52; CI, 1.11–2.07; *p* = 0.007) and after (OR, 1.51; CI, 1.10–2.07; *p* = 0.011) adjusting for clinical features including age, sex, BMI and diabetes (Table 3). After further adjusting for maximum NWI, the association of %volume of LRNC and stroke subtype still remained statistically significant (OR, 1.41; CI, 1.01–1.96; *p* = 0.041).

### Reproducibility

We found the intraclass correlation coefficient (ICC) for measuring morphology and compositions of carotid plaques was ranging from 0.81 to 0.91 for intra-observer agreement. For the intra-observer agreement, the Kappa value for identification of the presence of calcification, LRNC, and IPH was 0.91, 0.84, and 0.87, respectively.

For inter-observer agreement in measuring morphology and compositions of carotid plaques, the ICC was ranging from 0.80 to 0.89. For the inter-observer agreement in the presence of calcification, LRNC, and IPH, the Kappa value was 0.85, 0.83, and 0.86, respectively.

## Discussion

This study investigated the differences in the carotid plaque characteristics between patients with TIA and ischemic stroke using multi-contrast vessel wall MR. We found that stroke patients were more likely to have diabetes than patients with TIA. For the carotid plaque characteristics, stroke patients had significantly greater mean wall area, mean wall thickness, maximum NWI and %volume of LRNC in the carotid arteries compared with those with TIA before and after adjusting for age, sex, BMI and diabetes. After adjustment for maximum NWI, the association of %volume of LRNC and the type of ischemic events still remained statistically significant. Our results suggest that carotid plaques in stroke patients may be more vulnerable than those in TIA patients due to larger LRNC regardless of plaque burden.

In this study, our results showed that ischemic stroke patients had a significantly higher prevalence of diabetes compared with TIA patients, which is supported by previous studies. Sun et al. reported that patients with type 2 diabetes had more concomitant large perforating artery infarct and larger acute cerebral infarct size compared with those with no diabetes [14]. In addition, another study by Thiruvoipati et al. also showed that diabetes was associated with greater severity of cardiovascular disease and worse clinical outcomes compared to nondiabetics [15]. Diabetes may promote inflammation infiltration and larger LRNC formation in carotid plaques, leading to plaque rupture and larger emboli formation [16], which will be more likely to result in a subsequent ischemic stroke rather than a TIA.

We found that ischemic stroke patients had significantly greater maximum NWI compared with those with TIA. Additionally, no significant relationship but a tendency was found between degree of stenosis and the type of event in the present study. A histological study [17] showed that stenosis of ipsilateral carotid plaques from stroke patients were more severe compared to those with TIA. The investigators thought that ischemic stroke might be caused by intracranial vascular embolism due to the rupture of moderately or severer stenotic plaque compared with TIA or asymptomatic patients. In addition, patients with high-grade stenosis are more susceptible to cerebral ischemia than those with normal cerebral perfusion and more likely to experience a stroke [18].

In the present study, we found that carotid plaques in stroke patients had a larger proportion of LRNC compared with those in TIA patients. The different size of LRNC between carotid plaques in patients with TIA and stroke might be associated with differences in leaky microvasculature and active inflammation in plaques. van Hoof et al. [7] compared the differences in microvasculature of carotid plaques between patients with recent stroke and TIA and found a positive association of leaky plaque microvasculature with recent stroke compared to TIA. In addition, another histological study performed by Spagnoli [17] showed that carotid plaques in patients with ipsilateral ischemic stroke had higher inflammation infiltrate compared with patients with TIA (74.0% vs. 35.2%, *p* < 0.001). Therefore, in patients with ischemic stroke, the increasing microvasculature and inflammation infiltrate in carotid plaques might promote the necrotic core formation and increase the size of LRNC compared to those with TIA.

Previous studies have demonstrated that a large LRNC was a major feature of vulnerable atherosclerotic plaques [19, 20]. The rupture of carotid plaques with larger LRNC in stroke patients might produce showers of larger emboli and resulting in cerebral infarcts. In contrast, patients with TIA were suffered with smaller emboli and lodged in small branch of cerebral arteries [21]. Accordingly, our findings suggest that TIA may be more likely to be due to embolization of platelet aggregates, whereas stroke may be more likely to be due to embolization of atheromatous debris from ruptured plaques. Additionally, our results also indicate that carotid atherosclerotic plaques in ischemic stroke patients are more vulnerable compared with those with TIA, which might explain the question that why ischemic stroke patients had a higher risk of recurrent ischemic events than those with TIA. Furthermore, the present study can provide a direction to further investigate that the quantitative measurement of LRNC in carotid plaques might be used as an important marker of the severity of subsequent cerebrovascular events.

The present study has several limitations. Firstly, the sample size of the present study was small and further studies with large sample size are warranted. Secondly, there are many potential causes of ischemic stroke, such as cardiogenic embolus, intracranial atherosclerosis, carotid atherosclerosis, etc. Although many efforts were made in the present study to reduce the possibility that the ischemic stroke was from other sources except carotid atherosclerosis, it is still difficult to completely confirm the mechanism of ischemic event without the pathological confirmation [22]. Thirdly, the qualitative measurements of carotid plaque burden and compositions could be influenced by the partial volume effects induced by the two-dimensional MR imaging techniques in the current study, especially for the volume measurements of carotid plaques. Three-dimensional (3D) MR vessel wall imaging techniques which have been recently developed could minimize the partial volume effects and be used to make more accurate comparison of carotid plaque measurements [23, 24].

## Conclusions

Ischemic stroke patients had larger plaque burden and greater proportion of LRNC in symptomatic carotid plaques compared to those with TIA. This study suggests that ischemic stroke patients had more vulnerable plaques, which could provide additional perspective to clarity the mechanism of higher recurrence of ischemic events in stroke patients compared to those with TIA.

## Data Availability

The datasets used and/or analyzed during the current study are available from the corresponding author on reasonable request.

## Abbreviations

TIA: Transient ischemic attack
MRI: Magnetic resonance imaging
IPH: Intraplaque hemorrhage
LRNC: Lipid-rich necrotic core
NWI: Normalized wall index
CEA: Carotid endarterectomy
TOF: Time-of-flight
BMI: Body mass index
CA: Calcification
